# EEG findings in SERAC1‐related MEGD(H)EL syndrome

**DOI:** 10.1002/epd2.70312

**Published:** 2026-06-09

**Authors:** Apurva Patel, Dalila Lewis, Thomas Koch

**Affiliations:** ^1^ Department of Neurology Medical University of South Carolina Charleston South Carolina USA; ^2^ Department of Pediatric Neurology Medical University of South Carolina Charleston South Carolina USA

**Keywords:** generalized spike‐and‐wave discharges, multifocal spike‐and‐wave discharges, paroxysmal fast activity, SERAC1‐related MEGD(H)EL syndrome

SERAC1 (Serine active site‐containing protein 1) deficiency phenotype ranges from MEGD(H)EL (3‐methylglutaconic aciduria with deafness‐dystonia, [hepatopathy], encephalopathy, and Leigh‐like syndrome) to juvenile complicated spastic paraplegia and adult‐onset dystonia.^1^, ^2^ MEGD(H)EL syndrome affects multiple systems including neurological, gastrointestinal, cardiac, endocrine, and musculoskeletal.^1^, ^2^ Neurological manifestations include hypotonia, spasticity, seizures, dystonia, and moderate‐to‐severe intellectual disability.^1^, ^2^, ^3^ Seizures are recognized as a component of the MEGD(H)EL syndrome and have been documented in case reports and cohort studies.^1^, ^2^, ^3^ However, the detailed electroencephalographic (EEG) description remains limited in the published literature.^2^, ^4^, ^5^, ^6^ Here we present a genetically confirmed SERAC1‐related MEGD(H)EL syndrome patient who presented with new‐onset seizures and demonstrated distinctive EEG findings.

This is a 7‐year‐old female with SERAC1‐related MEGD(H)EL syndrome, due to homozygous four‐base duplication (nucleotides 1347–1350) in the SERAC1 gene. She was born small for gestational age at 39 weeks and 5 days via spontaneous vaginal delivery and developed severe hypoglycemia, metabolic acidosis and *Escherichia coli* sepsis within the first 24 h of life. Additional neonatal complications include direct hyperbilirubinemia and left portal vein thrombosis.

Her development was severely delayed; at 18 months, she was nonverbal and was unable to roll, sit, or hold her head up; at age 3, gastrostomy tube was placed for poor feeding and failure to thrive. Brain magnetic resonance imaging at age 1 demonstrated mild‐to‐moderate cerebellar volume loss, with symmetric bilateral signal abnormalities in the basal ganglia, the posterior limb of the internal capsule, and the anterior commissure. Laboratory evaluation at age 3 revealed severe metabolic acidosis, elevated plasma amino acids (alanine and glutamine). Subsequent genetic testing revealed a homozygous pathogenic variant in the SERAC1 gene. Despite mitochondrial supplementation regimen including Coenzyme Q10, thiamine, and biotin, she continued to demonstrate severe neurological impairment with inability to attain speech or motor skills.

At 6 years of age, she began experiencing seizure‐like events characterized by muscle stiffening and abnormal movements with loss of consciousness. An EEG was obtained and was notable for striking interictal abnormalities, though no seizure events were captured.

Background activity showed diffuse intermittent delta‐theta slowing with occasional periods 2–3 s of generalized background attenuation consistent with moderate‐to‐severe encephalopathy (Figure 1D). Spike‐and‐wave discharges (SWDs) were recorded from multiple regions of the brain including right temporal, right parietal, left posterior temporal and left frontal abundantly without regional predominance or lateralization (Figure 1A,B). No clear definite sleep architecture was present on EEG, despite clinical evidence of sleep. During sleep, generalized SWDs were recorded and were followed by generalized paroxysmal fast activity (PFA) consisting of runs of 20–25 Hz rhythmic activity (Figure 1C). Generalized SWDs were symmetrical and synchronous in both hemispheres.

**FIGURE 1:**
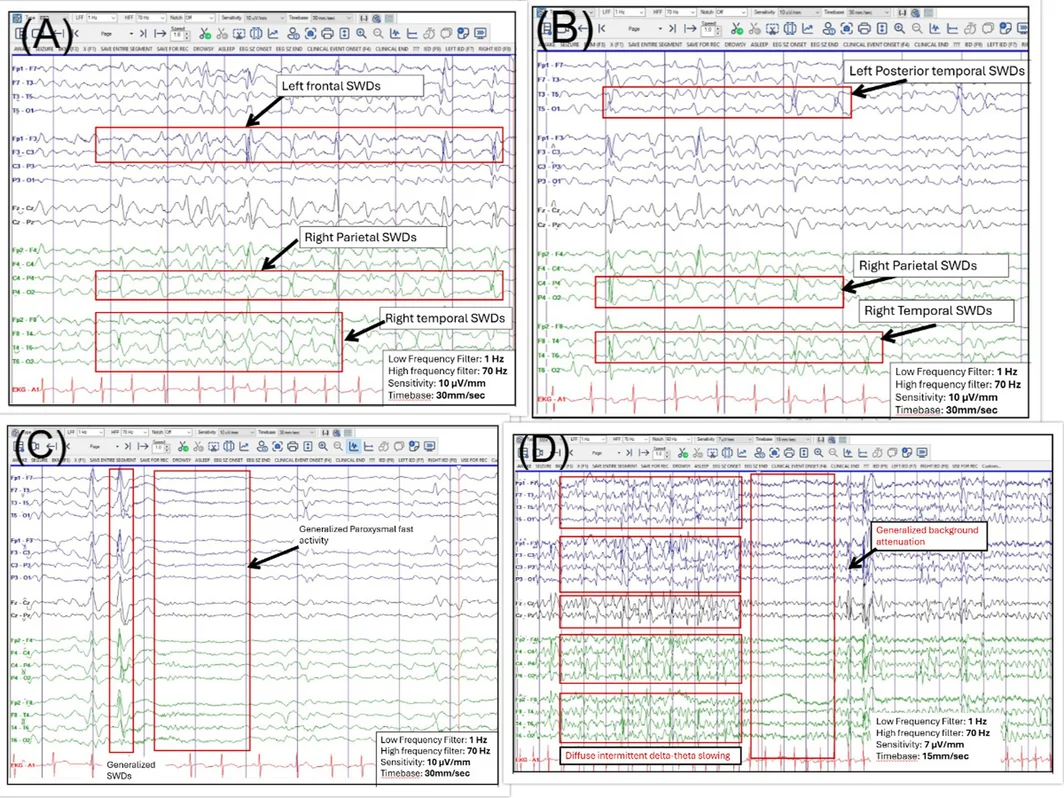
EEG findings in SERAC1‐related MEGD(H)EL syndrome. EEG segments are displayed using a longitudinal bipolar montage according to the international 10–20 system, with a high‐frequency filter of 70 Hz and a low‐frequency filter of 1 Hz. Sensitivity was set at 10 μV/mm in panels (A–C), and at 7 μV/mm in panel (D). (A, B) Multifocal SWDs (right temporal, right parietal, left posterior temporal and left frontal regions). (C) Generalized SWDs followed by 20–25 Hz PFA. (D) Diffuse delta‐theta slowing and a period of 2–3 s of generalized background attenuation. EEG, Electroencephalogram; MEGD(H)EL, 3‐methylglutaconic aciduria with deafness‐dystonia, [hepatopathy], encephalopathy, and Leigh‐like syndrome; SERAC1, Serine active site‐containing protein 1; SWDs, Spike‐and‐wave discharges; PFA, paroxysmal fast activity.

This case represents one of the first detailed characterizations of EEG findings in a patient with genetically confirmed SERAC1‐related MEGD(H)EL syndrome. Generalized PFA is defined as bursts of 15–25 Hz frontally dominant rhythmic activity, typically appearing during sleep or drowsiness, and is considered an interictal EEG marker of increased seizure susceptibility.^7^, ^8^ PFA is a pattern characteristic of developmental and epileptic encephalopathies (DEEs) such as Lennox–Gastaut syndrome and has been described in other genetic epilepsies. Its occurrence in SERAC1‐related MEGD(H)EL syndrome has not been previously reported, and this EEG pattern may represent an underrecognized feature of the disorder's electroclinical manifestations.^3^, ^4^, ^5^, ^6^, ^7^, ^9^

This clinical vignette is limited by its single‐patient design and the absence of captured seizures during the recording; long‐term monitoring might have offered stronger electroclinical correlation. Also, the patient has not undergone repeat EEGs to assess the temporal evolution of the abnormalities. Larger cohorts and longitudinal follow‐up studies would better define the prevalence and significance of PFA and other EEG patterns in SERAC1‐related MEGD(H)EL syndrome. Overall, these findings broaden the known electroclinical phenotype and highlight the value of sleep‐inclusive EEG analysis, while emphasizing the need for continued systemic EEG characterization to guide prognosis and management in this rare disorder.

## AUTHOR CONTRIBUTIONS

Apurva Patel drafted the manuscript. Dalila Lewis supervised the project. Thomas Koch was involved in the clinical management of the patient. All authors have read and approved the final version of the manuscript.

## FUNDING INFORMATION

The authors received no funding for this project.

## CONFLICT OF INTEREST STATEMENT

None of the authors have any conflict of interest to disclose.

**Test yourself**

1. What types of epileptic discharges were observed in this patient with SERAC1‐related MEG(H)EL syndrome?
  - Polyspike‐and‐wave discharges.
  - Multifocal and Generalized spike‐and‐wave discharges.
  - Temporal lateralized rhythmic delta activity.
  - Occipital slow spike‐and‐wave discharges.
2. Which EEG pattern characteristics of developmental and epileptic encephalopathies (DEEs) such as Lennox–Gastaut syndrome was seen in this patient with SERAC1‐related MEG(H)EL syndrome?
  - High‐frequency oscillations (HFO).
  - Hypsarrhythmia.
  - Generalized paroxysmal fast activity.
  - Direct current (DC) shifts.
3. Which of the following are the characteristics of Generalized paroxysmal fast activity?
  - Frontally dominant; increases in sleep.
  - Temporally dominant; increases in sleep.
  - Frontally dominant; decreases in sleep.
  - Posteriorly dominant; increases in sleep.

*Answers may be found in the* *Supporting Information*.

## Supporting information


Data S1:

Data S2:

## Data Availability

The data that support the findings of this study are available from the corresponding author upon reasonable request.
