# Supplementary material for: EEG findings in SERAC1‐related MEGD(H)EL syndrome
Source: Epileptic Disord. 2026 Jun 9;28(5):1719–22. doi: 10.1002/epd2.70312 (PMC13695482; doi:10.1002/epd2.70312)
Supplement: Supplementary file 1 [file EPD2-28-1719-s002.docx]

**TEST YOURSELF**

**Answers**

1. B

2. C

3. A
